# Anticoagulant therapy for nodular regenerative hyperplasia in a HIV-infected patient

**DOI:** 10.1186/1471-230X-10-6

**Published:** 2010-01-18

**Authors:** Florian Bihl, Filip Janssens, Francoise Boehlen, Laura Rubbia-Brandt, Antoine Hadengue, Laurent Spahr

**Affiliations:** 1 Department of Gastroenterology and Hepatology, University Hospitals of Geneva, Rue Gabrielle-Perret-Gentil 4, Geneva 1211, Switzerland; 2 Department of Hematology, Service of Angiology and Hemostasis, University Hospitals of Geneva, Rue Gabrielle-Perret-Gentil 4, Geneva 1211, Switzerland; 3 Department of Pathology, University Hospitals of Geneva, Rue Gabrielle-Perret-Gentil 4, Geneva 1211, Switzerland

## Abstract

**Background:**

Nodular regenerative hyperplasia (NRH) has been recently recognized as an emergent cause of liver disease in HIV-infected patients. NRH may cause non-cirrhotic portal hypertension with potentially severe consequences such as refractory ascites, variceal bleeding and hypersplenism. Obliteration of the small intrahepatic portal veins in association with prothrombotic disorders linked to HIV infection itself or anti-retroviral therapy seem to be the causes of NRH and thus the term HIV-associated obliterative portopathy has been proposed.

**Case Presentation:**

Here we describe a case of a HIV-infected patient with biopsy-proven NRH and listed for liver transplantation (LT) because of refractory ascites and repeated upper gastrointestinal bleedings. A transjugular intrahepatic portosystemic shunt was placed as a bridge to LT and did not improve liver function. However, anticoagulant therapy with low-molecular-weight heparin (LMWH) was associated with rapid improvement in the liver condition and allowed to avoid LT in this patient.

**Conclusions:**

Thus, this case underscores the relation between thrombophilia and HIV-associated NRH and emphasizes anticoagulant therapy as possible treatment.

## Background

Nodular regenerative hyperplasia (NRH) is a diffuse disorder of the liver characterized by nodular transformation of the hepatic parenchyma without fibrosis. NRH causes intra-hepatic non-cirrhotic portal hypertension (NCPH)[1]. Recently, several reports described HIV-infected patients with symptomatic NCPH revealing NRH [1-13]. Typical histopathological features of NRH include atrophic hepatocytes together with areas of hypertrophyic plates arranged in multilayer around the portal tract, producing nodules in the absence of significant fibrosis. The small portal veins are obliterated leading to an atrophy of the supplied acinus and to a subsequent hypertrophy of the adjacent acinus. Sinusoidal dilatation is secondary to an increased blood flow. The sum of these events has recently be described by Mallet et al. coining the term HIV-associated obliterative portopathy (HIV-OP) [10]. The prevalence of NRH is not well established because diagnosis is difficult on needle biopsies and patients are usually asymptomatic at early stages. Although the cause of NRH is not fully understood, it seems that NRH is secondary to HIV-OP and associated with an hypercoagulable state [10,14,15]. A number of diseases have been associated with NRH, in particular haematological disorders with thrombophilia including myeloproliferative or lymphoproliferative diseases [15,16] which can be found in more than half the patients presenting with NRH [14]. In addition, autoimmune diseases (e.g. systemic lupus erythematosus, rheumatoid arthritis, celiac disease, etc) and immune-suppression have been associated with NRH. The thrombophilia in HIV-OP is thought to be an acquired protein S deficiency with lower protein S levels and decreased activity [10].

Treatment for NRH would ideally include correction of triggering factors to prevent disease extension. However, therapy of NRH is usually limited to the treatment of complications of portal hypertension, i.e. beta-blockers, variceal ligation and/or portosystemic shunts (TIPS). Moreover, liver transplantation (LT) has been performed in cases with severe portal hypertension and/or liver failure [17,18]. Interestingly a recent case series of LT in HIV-infected patients with NRH described an overall good outcome [9].

Here we describe a patient with HIV infection who developed severe intrahepatic non-cirrhotic portal hypertension due to NRH with a decreased proteins S level and recurrent variceal bleeding, refractory ascites and cachexia despite TIPS placement. The patient was listed for liver transplantation. While awaiting LT, anticoagulant therapy with low-molecular-weight heparin (LMWH) allowed spectacular and sustained improvement of the patient's condition, which led to remove the patient from the waiting list. To our knowledge this is the first case of HIV-related NRH that was successfully treated with anticoagulant treatment avoiding liver transplantation.

## Case Presentation

In August 2005 a 43-year old woman with known HIV infection since 1998 presented in the emergency department with post-prandial abdominal pain and important weight loss (10 kg) over 4 months. HIV infection was treated since 2000 with a HAART including didanosine, lamivudine and abacavir. At admission her absolute CD4 count was 279/mm^3 ^and HIV viremia 270,000 copies/ml. Diffuse lymphoadenopathy, splenomegaly and ascites were observed associated with biochemical abnormalities of the liver parameters: aspartate aminotransferase (AST) and alanine aminotransferase (ALT) were 1.5 times upper normal range, alkaline phosphatase (AP) twice and gamma-glutamyltransferase (γGT) four times above normal values. There was neither alcohol intake nor toxic exposure; screening was negative for viral hepatitis (HAV, HBV, HDV, HCV and HEV), CMV and EBV, autoimmune hepatitis (antinuclear antibodies, antiactin antibodies and total serum immunoglobulins), Wilson's disease, hemochromatosis and schistosomiasis. A thoraco-abdominal CT scan showed mediastinal and axillar lymphadenopathies, pulmonary nodules and a heterogeneous liver parenchyma with signs of portal hypertension (esophageal varices, ascites and splenomegaly). A transjugular liver biopsy revealed a slightly abnormal portal pressure gradient (8 mmHg wedge gradient) and the histology described normal parenchymal architecture with a minimal portal fibrosis and sinusoidal infiltration of mainly T lymphocytes (CD3+). A lymphoproliferative disease was ruled out by flow cytometry of peripheral blood lymphocytes, intranodal lymphocytes (axillar lymphnode biopsy) and of liver infiltrating lymphocytes. At this point our hypothesis pointed towards a miliary tuberculosis with lung and peritoneal involvement. In the ascites fluid the polymorphonuclear count was low (PMN, <250 cells/mm^3^) as also the albumin (10 gr/l) and protein level (9 gr/l) with a serum-to-ascites albumin gradient (SAAG) of 2.6 pointing towards portal hypertension. However, the cultures of the ascites fluid tested positive for *M. tuberculosis *and treatment was started with rifampin, isoniazid (INH) and ethambutol for six months and thereafter bitherapy with INH and rifampin (based on the antibiogram) for additional seven months. An upper gastrointestinal endoscopy evidenced grade I esophageal varices. For the viral breakthrough of HIV, HAART was changed to tenofovir, efavirenz, atazanavir and ritonavir. The patient was dismissed from the hospital but presented seven, eight, ten and 12 months later severe esophageal variceal bleedings treated with endoscopic band ligation and slerotherapy and beta-blockers (propanolol).

**Figure 1:**
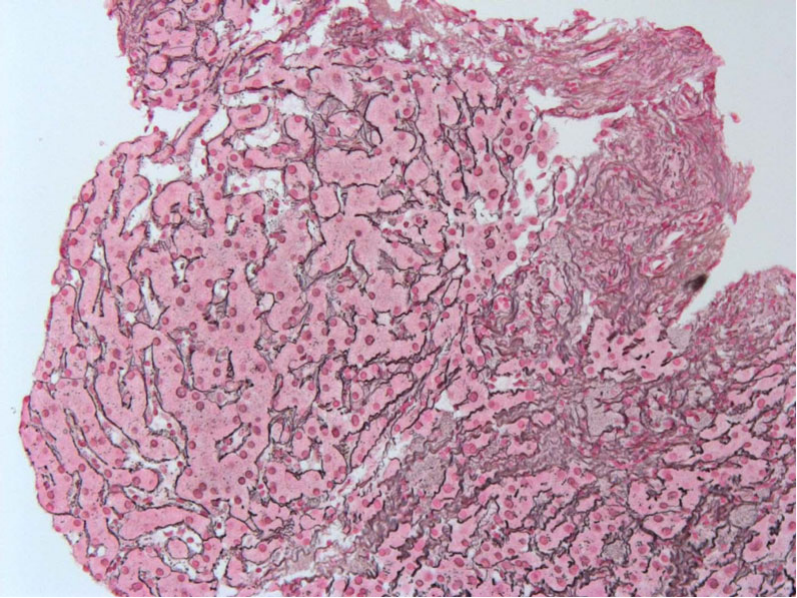
**A liver biopsy performed in September 2006 shows in the reticulin stain an altered architecture with enlarged hepatocytes arranged in small nodules (×400)**.

In September 2006 she was admitted again for bilateral leg swelling and important ascites with very low proteins (7 gr/l, serum albumin 30 gr/l, serum-to-ascites albumin gradient SAAG of 2.3). Liver function tests were still abnormal: AST and ALT 1.5 ULN, AP 2× ULN, γGT 4× ULN and diminisched Factor V (50%). An abdominal CT scan and MRI showed large volume ascites, splenomegaly and portal-systemic collaterals. The portal vein was patent. A second transjugular liver biopsy revealed again minimal portal fibrosis without significant inflammation. On the reticulin stain a typical nodular rearrangement of the parenchyma with atrophic periportal and regenerative centrolobular plates was compatible with NRH (Figure 1). In addition, fibrous material was occluding portal veins in several portal tracts pointing towards HIV-associated obliterative portopathy.

**Figure 2:**
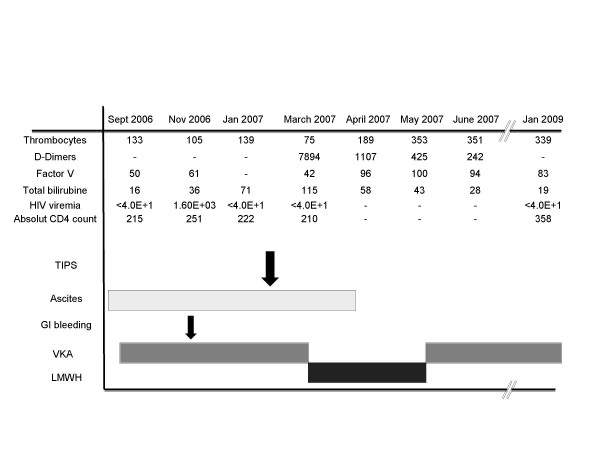
**Evolution of liver function, clinical parameters and selected parameters of haemostasis were shown**. Please note the changes at the time of the switch from VKA to LMWH.

Hence, in the context of HIV infection, we suspected a hypercoagulable state with micro-thrombosis in the distal portal veins causing NRH. A thrombophilia work-up (lupus anticoagulant, anticardiolipin and anti-β2-glycoprotein I antibodies, factor II G20210A mutation, factor V Leiden mutation, anti-thrombin, protein C and protein S) showed low level of free protein S antigen (37% normal > 55%). An anticoagulant treatment with vitamin K antagonists (VKA, acenocoumarol) aiming at an INR of 2.0-3.0 was introduced. However, the anticoagulant treatment with VKA was difficult to regulate with highly variable INR, which was repeatedly infra-therapeutic. In November 2006 she presented another variceal bleeding (with an INR at 1.7) controlled by band ligation, ascites and progressive cachexia (BMI 15.6). HIV infection was again uncontrolled and treatment was changed to tenofovir, emtricitabin, lopinavir and ritonavir. Thus, in February 2007 the patient was listed for LT with a MELD score of 14. To bridge until LT a TIPS was placed. Four weeks after the TIPS placement, the anticoagulant treatment with VKA was still extremely difficult to manage with unsatisfactory INR levels. Low platelets counts, factor V level, and fibrinogen (2.1 g/l) together with high D-dimers were suggestive of a persistent consumption coagulopathy and an ongoing thrombotic process. For these reasons, anticoagulant treatment was switched to LMWH in March 2007 for the next two months. With this treatment, the factor V levels increased, D-dimers normalized and fibrinogen increased (to 6.5 g/l); this was all highly suggestive of efficient anticoagulant treatment (resumed in Figure 2). In May 2007 anticoagulant treatment was switched back to VKA and INR could be maintained between 2 and 3. In the following months ascites and lower leg edema disappeared, the nutrition state improved considerably, and finally the patient was removed from the LT waiting list. While writing this report the patient was well with a BMI of 21.

## Conclusion

Serious NRH complicating HIV infection has been recently reported by several groups [1-13]. Severe portal hypertension with variceal bleedings and refractory ascites can precipitate cachexia and threaten life of these patients. So far HIV-related NRH portal hypertension was treated symptomatically or referred for LT. Here we show that clinical improvement of NRH with LMWH therapy is possible. This case fits to the recent observations of Mallet et al. describing an HIV-associated obliterative portal venopathy (HIV-OP) associated with a thrombophilia. Ongoing thrombosis in the distal portal veins in our patient is sustained by different observations: First, our patient presented with a low level of protein S during the thrombophilia workup pointing to a coagulation disorder that has been found in the majority of patients presenting with NRH (protein S or C deficiency and/or anti-cardiolipin antibodies) [10,14]. Second, the high D-dimer levels, low platelets and low fibrinogen levels were suggestive of a constant thrombotic process with activation of the coagulation system and consumption of coagulation factors. Indeed, the fibrotic tissue occluding the portal veins is probably consecutive to thrombosis. Third, the treatment with VKA and the placement of a TIPS did not correct impaired coagulation balance. However, the introduction of a therapy with LMWH (enoxaparin) was associated with clear-cut biochemical and clinical improvement. Major recovery of the general condition and absence of ascites paralleled the decrease of D-dimer, the increase of fibrinogen and platelets. Indeed, LMWH seems to be superior to VKA for the treatment of venous embolism as it has been shown in patients with cancer [19]

Different hypotheses have been evoked as causes for NRH in HIV-infected patients. Underlying diseases like autoimmune disorders, malignancies or active viral infections such as HIV may create an imbalance of pro- and anti-thrombotic factors. Recently, Mallet et al. showed that protein S levels are not only often low in patients with HIV-associated NRH but the functionality of the protein S is decreased due to increased levels of antiprotein S IgG. Their data suggest, that the antiprotein S IgG were acquired during longstanding HIV infection and contribute to the obliterative portal venopathy [10]. Although not vasculotropic, HIV exposes the vascular endothelium constantly to viral stimuli such as viral proteins upon lysis of infected CD4 T cells and viral-induced pro-inflammatory cytokines. The latter increase the endothelial cell permeability and facilitate monocyte invasion and activation in the vessel wall [20]. Thus, HIV directly alters the endothelium homeostasis and leads to clotting dysfunction contributing to thrombotic complications in the arterial and venous system [21]. The direct endothelial toxicity triggered by the virus has been already evoked in patients with HIV-associated pulmonary hypertension [22]. Moreover, there is clinical and *in vitro *evidence that ART may have a toxic effect on the endothelium and cause vascular dysfunction [6,11,20,21,23]. Even if most cases of thromboembolic events in HIV-infected have been associated with protease inhibitors, the nucleoside-reverse-transcriptase inhibitor (NRTI) didanosin was associated in several studies with NCPH and NRH [2,12]. A recent case-control study from the Swiss HIV cohort study showed that among individual antiretroviral drugs, didanosin exposure was a clear risk factor for development of NCPH [12]. Indeed, didanosin is thought to lead to a mitochondrial damage in endothelial cells [24]. In any case, our patient was treated with Didanosin for five years before she developed symptoms of portal hypertension and maybe the switch from didanosine to other ART components has also contributed in the clinical improvement.

Taken together, this case underscores once again the relation between thrombophilia and HIV-associated NRH and emphasizes a potential beneficial role of anticoagulant therapy. A continuously activated prothrombotic state was difficult to interrupt with VKA and only after the introduction with LMWH the thrombotic markers ameliorated. Although potentially dangerous because major bleedings can occur, we think that anticoagulant treatment with LMWH could play a role in the prognosis in NRH as suggested by this case and may avoid, at least in certain cases, liver transplantation. However, this hypothesis should be evaluated in controlled trial analyzing anticoagulant therapy with AVK and LMWH.

## Abbreviations

HIV: human immuno deficiency virus; NRH: nodular regenerative hyperplasia; NCPH: noncirrhotic portal hypertension; HIV-OP: HIV-associated obliterative portal venopathy; AVK: anti-vitamin K agent; LMWH: low-molecular-weight heparin; TIPS: transjugular intrahepatic porto-systemic shunt; LT: liver transplantation; AST: aspartate amino transferase; ALT: alanine amino transferase; AP: alkaline phophatase; gGT: gamma glutamyl transpeptidase

## Competing interests

There was no funding for this case report. All authors declare that they have no conflict of interest in relation to this manuscript

## Authors' contributions

FBi, FBo, LRB, AH and LS were directly involved in the clinical management of the patient. FBi, FJ, collected the data and drafted the manuscript together with FBo, AH and LS. LRB revised the manuscript critically and added substantial intellectual content. All authors read and approved the final manuscript.

## Pre-publication history

The pre-publication history for this paper can be accessed here:

http://www.biomedcentral.com/1471-230X/10/6/prepub
